# Physiologic dead space is independently associated with mortality and discharge of mechanically ventilated patients with COVID-19 ARDS: a retrospective study

**DOI:** 10.1038/s41598-023-31999-6

**Published:** 2023-04-07

**Authors:** Maximiliano Mollura, Francesca Baroncelli, Giulia Mandelli, Giovanni Tricella, Gary E. Weissman, Daniele Poole, Pietro Caironi, Leo Anthony Celi, Riccardo Barbieri, Stefano Finazzi

**Affiliations:** 1 Department of Electronic, Information and Bioengineering, Politecnico di Milano, Milan, Lombardia Italy; 2 Department of Anesthesia and Intensive Care, San Giovanni Bosco Hospital-ASL Città di Torino, Turin, Piemonte Italy; 3 Department of Medical Epidemiology, Mario Negri Institute for Pharmacological Research IRCCS, 24020 Ranica, Lombardia Italy; 4 Palliative and Advanced Illness Research (PAIR) Center and Pulmonary, Allergy and Critical Care Division, University of Pennsylvania Perelman School of Medicine, Philadelphia, PA USA; 5 Operative Unit ofAnesthesia and Intensive Care Unit, S. Martino Hospital, Belluno, Veneto Italy; 6 Department of Anesthesia and Critical Care, Azienda Ospedaliero-Universitaria S. Luigi Gonzaga, Orbassano, Piemonte Italy; 7 Department of Oncology, University of Turin, Turin, Piemonte Italy; 8 Laboratory for Computational Physiology, Massachusetts Institute of Technology, Cambridge, MA USA; 9 Division of Pulmonary, Critical Care and Sleep Medicine, Beth Israel Deaconess Medical Center, Boston, MA USA; 10 Department of Biostatistics, Harvard T.H. Chan School of Public Health, Boston, MA USA

**Keywords:** Translational research, Viral infection, Respiratory distress syndrome

## Abstract

Physiologic dead space is a well-established independent predictor of death in patients with acute respiratory distress syndrome (ARDS). Here, we explore the association between a surrogate measure of dead space (DS) and early outcomes of mechanically ventilated patients admitted to Intensive Care Unit (ICU) because of COVID-19-associated ARDS. Retrospective cohort study on data derived from Italian ICUs during the first year of the COVID-19 epidemic. A competing risk Cox proportional hazard model was applied to test for the association of DS with two competing outcomes (death or discharge from the ICU) while adjusting for confounders. The final population consisted of 401 patients from seven ICUs. A significant association of DS with both death (HR 1.204; CI 1.019–1.423; *p* = 0.029) and discharge (HR 0.434; CI 0.414–0.456; *p* $< 0.001$) was noticed even when correcting for confounding factors (age, sex, chronic obstructive pulmonary disease, diabetes, PaO$_{2}$/FiO$_{2}$, tidal volume, positive end-expiratory pressure, and systolic blood pressure). These results confirm the important association between DS and death or ICU discharge in mechanically ventilated patients with COVID-19-associated ARDS. Further work is needed to identify the optimal role of DS monitoring in this setting and to understand the physiological mechanisms underlying these associations.

## Introduction

Endothelial inflammatory damage and pulmonary microvascular dysfunction, resulting in microthrombosis and pulmonary vascular perfusion defects, are prominent features of acute respiratory distress syndrome (ARDS) associated with Coronavirus disease 2019 (COVID-19)^1,2^. Although the contribution of pulmonary microthrombosis to the pathophysiology of ARDS is long-known^3,4^, the role of microangiopathy and and dysregulated lung perfusion appears even more relevant in COVID-19-associated ARDS as compared to other infectious causes of ARDS^5,6^.

The immune-mediated thrombosis of alveolar capillaries may be implicated in the high physiological dead space reported in patients with COVID-19-associated ARDS^7,8^, which appears unrelated to the compliance of the respiratory system (C$_{\textrm{RS}}$)^9^.

An elevated dead space is a well-established independent predictor of death in patients with ARDS of different etiologies^10–12^. The Enghoff’s modification of Bohr’s equation^13^, which can be further simplified to the end-tidal-to-arterial carbon dioxide tension ratio (EtCO$_{2}$/PaCO$_{2}$)^14^, has been used to calculate the physiologic dead space (DS), while also providing a global measure of the severity of impairment in gas exchange, for being influenced by intrapulmonary shunt, diffusion impairment and ventilation/perfusion heterogeneity in addition to dead space itself^15^.

Physiologic dead space can also be estimated through the ventilatory ratio (VR)^16^, the corrected minute ventilation^17^ or a modified Harris–Benedict equation^18^. Although they can not be used interchangeably^19^, all these indices have been used as direct or surrogate measures of physiologic dead space in ARDS, whose increment is consistently associated with an increased risk of death^14,20–23^.

In a recent secondary analysis of a retrospective national study, the PRoVENT-COVID^24^, patients with COVID-19-associated ARDS dying within 28 days since the beginning of mechanical ventilation also showed signs of a significantly increased physiologic dead space, both at baseline and in the first 3 days since intubation, as compared to survivors. Interestingly, the trend in physiologic dead space estimations significantly differed over time between survivors and non-survivors, suggesting that dynamic changes in the estimates of dead space during the course of the Intensive Care Unit (ICU) stay may be more informative than single measures at the very beginning of mechanical ventilation.

Here, we hypothesize that the trend of physiologic dead space over time is independently associated with mortality or discharge from the Intensive Care Unit in mechanically ventilated patients with COVID-19-associated ARDS.

## Results

### Cohort selection

**Figure 1:**
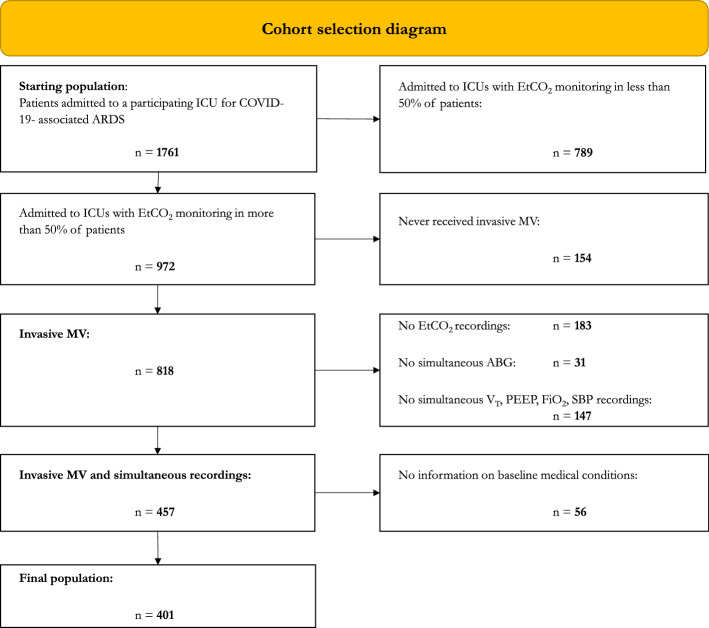
Cohort selection diagram. *ABG* arterial blood gas, *ARDS* acute respiratory distress syndrome, *EtCO*$_{2}$ end-tidal partial pressure of carbon dioxide (in mmHg), *FiO*$_{2}$ fraction of inspired oxygen, *ICU* intensive care unit, *MV* mechanical ventilation, *PEEP* positive end-expiratory pressure (in cmH$_{2}$O), *SBP* systolic blood pressure, *V*$_{\textrm{T}}$ set or measured tidal volume (in mL).

A flow chart showing the effect on the population size of the different selection steps is depicted in Fig. 1. The starting population consisted of 1761 patients admitted to the participating ICUs for COVID-19-associated ARDS between February 21st, 2020 and March 31st, 2021. The study population was composed mainly of men (1308, 74%) with a median age of 66 years (IQR, 58–73 years) and showed an ICU mortality of 33% (587) (Table 1). The median length of stay in the ICU was 10 days (IQR, 4–19 days), and about half of the patients were discharged from the ICU. The remaining 16% of the subjects was still in the ICU on 31st March, 2021: these patients were right-censored.

**Table 1:** Demographic variables and outcomes for the selected populations.

	Starting population (n = 1761)	ICUs with EtCO$_{2}$ $^{\ddag }$ (n = 972)	Mechanical ventilation (n = 818)	No mechanical ventilation (n = 154)	Final population (n = 401)
Sex (% females)	453 (25.7)	232 (23.9)	193 (23.6)	39 (25.3)	95 (23.7)
Age, years	66 (58–73)	65 (56–72)	66 (58–72)	63 (50–73)	66 (59–71)
ICU LOS, days	10 (4–20)	11 (5–22)	14 (7–25)	3 (2–5)	18 (10–30)
Dead (%)	587 (0.33)	278 (0.29)	266 (0.33)	12 (0.08)	150 (0.37)
Discharged (%)	884 (0.5)	467 (0.48)	353 (0.43)	114 (0.74)	163 (0.41)
Censored (%)	290 (0.16)	227 (0.23)	199 (0.24)	28 (0.18)	88 (0.22)

Data are median (interquartile range) or numbers (%). Percentages may not total 100 because of rounding. $^{\ddag }$Refers to Intensive Care Units which recorded EtCO$_{2}$ in more than 50% of patients. *EtCO*$_{2}$ end-tidal partial pressure of carbon dioxide, *ICU* intensive care unit, *LOS* length of stay.

After filtering out the centers that did not measure EtCO$_{2}$ in at least 50% of the admitted patients, the resulting population of 972 subjects (55%), admitted to seven ICUs from different Italian hospitals, showed similar characteristics to the initial one (Table 1).

A further step excluded 154 patients who never needed to receive invasive mechanical ventilation during their ICU stay, and for whom DS could not be measured. These patients predictably showed a more benign course of the disease, with a shorter length of stay (LOS) in the ICU and higher discharge rates when compared to the 818 subjects who needed invasive mechanical ventilation via an endotracheal tube or a tracheostomy.

Among the 818 patients who received invasive mechanical ventilation, 361 were further excluded from the analysis, either because the required measures for DS estimation (EtCO$_{2}$ or PaCO$_{2}$) were not recorded in the EHR or because simultaneous (i.e., within 1 h) recordings of physiological variables (SBP, V$_{\textrm{T}}$, PEEP, PaO$_{2}$/FiO$_{2}$, EtCO$_{2}$, PaCO$_{2}$) were not available.

Finally, 56 patients were excluded because of missing information about any baseline medical conditions (irrespective of their presence). Table S1 in the Supplementary material provides detailed information about the demographics and outcomes of excluded patients.

The final population then consisted of 401 patients. Among these, 150 (37%) died, 163 (41%) were discharged from the ICU and 88 (22%) did not experience the outcome before 31st March 2021. The subjects who died within 7 days since the last available set of recordings were 119 (79.3%) with a median (IQR) time to event of 18 (7–57) h from admission, whereas 71 patients (43.6%) were discharged from the ICU, showing a median (IQR) time to event from admission of 100 (60–136) h.

**Table 2:** Pre-existing conditions in selected populations.

	Starting population (n = 1567)	MV$^{\dag }$ (n = 715)	No MV$^{\ddag }$ (n = 137)	Final population (n = 401)	Dead (n = 150)	Discharged (n = 163)	Censored (n = 88)
None	239 (15.3)	107 (15.0)	29 (21.2)	63 (15.7)	14 (9.3)	33 (20.2)	16 (18.2)
Hypertension	905 (57.8)	429 (60)	73 (53.3)	236 (58.9)	104 (69.3)	81 (49.7)	51 (58.0)
Obesity	477 (30.4)	203 (28.4)	32 (23.4)	126 (31.4)	46 (30.7)	55 (33.7)	25 (28.4)
Type II DM, not on insulin	260 (16.6)	106 (14.8)	19 (13.9)	58 (14.5)	24 (16.0)	27 (16.6)	7 (8.0)
Type II DM, on insulin	117 (7.5)	51 (7.1)	6 (4.4)	25 (6.2)	10 (6.7)	5 (3.1)	10 (11.4)
Type I DM	11 (0.7)	2 (0.3)	2 (1.5)	1 (0.2)	0 (0)	1 (0.6)	0 (0)
COPD	133 (8.5)	53 (7.4)	28 (8.9)	25 (6.2)	7 (4.7)	13 (8.0)	5 (5.7)
COPD, moderate	114 (7.3)	48 (6.7)	9 (6.6)	23 (5.7)	6 (4.0)	12 (7.4)	5 (5.7)
COPD, severe	19 (1.2)	5 (0.7)	3 (2.2)	2 (0.5)	1 (0.7)	1 (0.6)	0 (0)
Asthma	65 (4.1)	26 (3.6)	3 (2.2)	14 (3.5)	5 (3.3)	7 (4.3)	0 (0)
Restrictive lung disease	6 (0.4)	3 (0.4)	0 (0)	2 (0.5)	0 (0)	2 (1.2)	0 (0)
Myocardial infarction	131 (8.4)	65 (9.1)	13 (9.5)	31 (7.7)	16 (10.7)	12 (7.4)	3 (3.4)
Arrhythmia	106 (6.8)	38 (5.3)	10 (7.3)	20 (5.0)	11 (7.3)	6 (3.7)	3 (3.4)
CHF (NYHA class 2 or 3)	40 (2.6)	13 (1.8)	6 (4.4)	4 (1.0)	2 (1.3)	1 (0.6)	1 (1.1)
Cancer, not metastatic	54 (3.4)	25 (3.5)	2 (1.5)	13 (3.2)	6 (4.0)	7 (4.3)	0 (0)
Hematological disorders	18 (1.1)	9 (1.3)	0 (0)	5 (1.2)	2 (1.3)	1 (0.6)	2 (2.3)
Autoimmune diseases	31 (2.0)	12 (1.7)	5 (3.6)	8 (2.0)	4 (2.7)	3 (1.8)	1 (1.1)
CKD, moderate or severe	80 (5.1)	33 (4.6)	8 (5.8)	15 (3.7)	6 (4.0)	8 (4.9)	1 (1.1)
CKD, end-stage	7 (0.4)	1 (0.1)	2 (1.5)	1 (0.2)	1 (0.7)	0 (0)	0 (0)
Cerebrovascular disease	54 (3.4)	26 (3.6)	7 (5.1)	13 (3.2)	7 (4.7)	4 (2.5)	2 (2.3)
Hemiplegia or paraplegia	13 (0.8)	8 (1.1)	2 (1.5)	5 (1.2)	3 (2.0)	1 (0.6)	1 (1.1)
Neuromuscular disease	11 (0.7)	4 (0.6)	3 (2.2)	0 (0)	0 (0)	0 (0)	0 (0)

Data are number (%). $^{\dag }$MV indicates patients who received invasive mechanical ventilation (MV), via endotracheal intubation or tracheostomy, during their stay in the Intensive Care Unit. $^{\ddag }$No MV indicates patients who never received invasive mechanical ventilation during their stay. *CHF* chronic heart failure, *CKD* chronic kidney disease, *COPD* chronic obstructive pulmonary disease, *DM* diabetes mellitus, *NYHA* New York functional classification for heart failure.

Table 2 shows the baseline comorbidities on ICU admission for the starting and the final population. As for the outcomes, the prevalence of the main comorbidities is comparable between the starting and the final populations. Additional information about the distribution of pre-existing medical conditions in patients who were excluded from the analysis is reported in Table S2 of the supplementary material. All patients received a moderate-to-deep level of sedation. Propofol was administered to most patients as the main sedative agent, along with other sedatives like midazolam or dexmedetomidine; the majority of patients (93%) also received an infusion of neuromuscular blocking agents. Summary statistics for physiologic measurements in the subjects who died, who were discharged from the ICU, or subjects who were censored are reported in the supplementary material in Table S3. Trends of the PaO$_{2}$/FiO$_{2}$ and DS over time are visually displayed in Figure S1 of the supplementary material.

**Figure 2:**
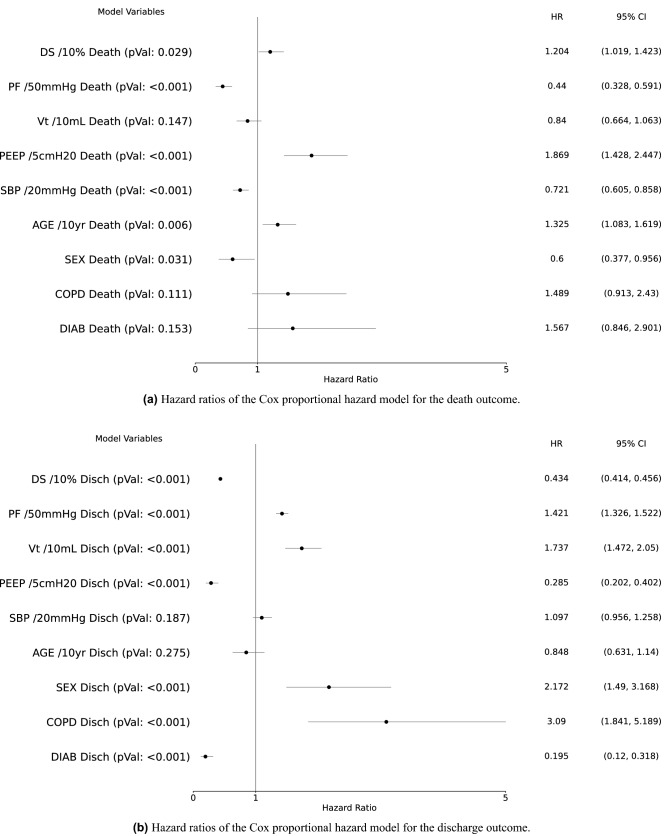
Hazard ratios (HR), confidence intervals (CI) and p values for each increment of selected variables with respect to the death (**a**) and the discharge (**b**) outcome at 7 days since the last available set of physiological data. Numbers alongside each continuous variable indicate the scaling factors for computing hazard ratios. *AGE* age in years, *COPD* presence or absence of chronic obstructive pulmonary disease, *DIAB* presence or absence or diabetes mellitus, *PEEP* positive end-expiratory pressure, in cmH$_{2}$O, *PF* ratio of arterial oxygen partial pressure (PaO$_{2}$, in mmHg) to fractional inspired oxygen (FiO$_{2}$), *SBP* systolic blood pressure in mmHg, *SEX* patient’s sex (females = 1, males = 0), *DS* physiologic dead space estimated using the Enghoff’s modification of the Bohr’s Equation, *Vt* tidal volume, in mL.

### Competing risk extended Cox model

Figure 2 shows the hazard ratios (HRs) for the competing risk Cox proportional hazard model.

More specifically, a 50 mmHg higher PaO$_{2}$/FiO$_{2}$ determined a significant 56% reduction in the hazard of death, and an increase of the hazard of discharge from the ICU (42%). On the contrary, a 10% higher DS was associated with a 20.4% increase in the HR of death and a 56.6% decrease in the hazard of discharge. A positive variation in SBP 20 mmHg was associated with a 28.1% reduction in the hazard of death. Each increment of 5 cmH$_{2}$O in PEEP was associated with a 86.9% increase in the hazard of death and a 71.5% reduction in the hazard of discharge. A V$_{\textrm{T}}$ variation of +10mL showed a significant 73.7% increase in the discharge hazard. A 10-year increase in age was associated with a 32.5% increase in the hazard of death. The presence of COPD and diabetes determined a 209% increase and 80.5% decrease in the discharge hazard, respectively. Female sex was associated with a 40% reduction in the hazard of death and a 117.2% increase in the hazard of discharge. The results obtained with standardized variables showed a 71% reduction in standardized hazard of death for higher PaO$_{2}$/FiO$_{2}$, which resulted to be larger than the increase in the standardized hazard of death due to a higher DS (31%).

Conversely, the standardized hazard of discharge for a decrease in DS (71%) was comparable with the standardized hazard of discharge observed for an increase in PaO$_{2}$/FiO$_{2}$ (71%).

Coefficients, hazard ratios, and p values of the model with standardized variables are available in Table S4 in the supplementary material. The likelihood ratio test between the model with and without the DS variable showed a significant difference (p $<0.001$).

## Discussion

The proposed competing risk extended Cox proportional hazard model for time-dependent covariates showed that in patients with COVID-19-associated ARDS, after correcting for the confounding effects related to ongoing treatments (V$_{\textrm{T}}$, PEEP), oxygenation indices (PaO$_{2}$/FiO$_{2}$), hemodynamic status (SBP), age, sex or relevant comorbidities (diabetes, COPD), an increase in DS is a significant predictor of death or reduced probability of discharge from the ICU within 7 days since the last available measure of COVID-19 patients. This result strengthens the significant role of DS as a global measure of gas exchange impairment in critically ill patients with ARDS, with added prognostic information to conventional markers of severity of ARDS, such as the PaO$_{2}$/FiO$_{2}$ ratio.

The estimator we propose (the dead space estimated according to the modified Bohr equation, based on the EtCO$_{2}$/PaCO$_{2}$ ratio), is easy to collect and focuses on physiological quantities directly measured from the patient.

The use of capnography in the ICU and the monitoring of dead space estimates have been long advocated^13,25^, given their role as a safety measure (assessment of endotracheal tube placement), as a marker of the adequacy of ventilation and global perfusion, and their prognostic role in specific situations, such as ARDS.

However, they still tend to be inconsistently monitored or assessed. In resource-limited situations, such as a pandemic, not only volume capnography but also simple time capnography may not be widely applied due to the lack of sufficient instrumentation, which could at least partly explain why a large number of mechanically ventilated patients did not have EtCO$_{2}$ measurements. In other instances, EtCO$_{2}$ might have been measured but not recorded in the EHR.

In our study, the time-series of relevant physiologic variables during the course of the ICU stay were used to predict death or discharge from the ICU within 7 days since the last measure. The prediction length of the outcome window was methodologically constrained by the need to fulfill the hypothesis of the proportional hazards assumption in Cox regression, which would have suffered by the use of a wider observation window for the selected outcomes. However, given the high variability of the respiratory and hemodynamic status of critically ill patients over time, which could be influenced by multiple intercurring events (e.g., a new hospital-acquired infection) and clinician interventions, the use of a 7-day window is also considered clinically appropriate.

Although corrected minute ventilation was found to be significantly associated with in-hospital mortality of patients admitted in the ICU due to COVID-19-induced ARDS^17,23^, other research focusing on a broader set of dead space estimators^24^ showed that dead space estimates at the onset and in the first day of mechanical ventilation for COVID-19-associated ARDS were not independent predictors of death at 28 days from the institution of invasive ventilation. However, while dead space estimates were not significantly different between survivors and non-survivors at the beginning and on the first day of mechanical ventilation, significant differences were found in the following days. In our analysis, we found a significant difference in the DS and PaO$_{2}$/FiO$_{2}$ ratios between survivors and non-survivors from the first recorded set of physiologic variables, that was maintained through the ICU stay to the last recorded set of variables. Our findings are in line with previous reports who showed an elevated dead space fraction in critically ill patients dying from COVID-19-associated-ARDS^9,26^, possibly because of a dysregulated endothelial activation promoting pulmonary microthrombosis^1,5^.

The proposed model and study design do not infer a causal relationship between the variable under study and the competing events, nor try to describe in detail the complex relationships between single ventilatory variables in the context of the altered respiratory physiology of COVID-19-associated ARDS. Rather, our study answers to a broader question about the observed association of the dead space estimation with ICU outcomes, when correcting for relevant confounders derived from comorbidities and major ventilatory and hemodynamic variables.

## Study limitations

Our study has several limitations. First, the retrospective nature of the study limits the availability of data for the analysis to those recorded in the electronic health record. Data availability may also depend on local clinical practice as well as current clinical and legal guidelines at the time of collection. Furthermore, the quality of the recorded data in retrospective studies might also be affected, as data have not been collected for research purposes. A second important limitation is that physiologic dead space was estimated using routinely available information in the EHR and not measured with the use of a gold-standard technique such as volumetric capnography. Indeed, blood gas analyses and time capnography have their own limitations, including sample contamination, sample collection mishap, tube positioning and obstruction, among others^27,28^. A third limitation is that patient status was assumed to be constant within a 1-h window for each set of measurements, thus implying that any delay between measurements belonging to the same time window cannot be considered in profiling the ventilatory and hemodynamic status of the patient.

## Conclusion

The present study expands and strengthens the well-known role of dead space monitoring, already observed in classical ARDS patients, to COVID-19-associated ARDS. The proposed modeling approach allows for the evaluation of the dynamic evolution of physiologic data from mechanically ventilated patients and specifically explores the association between dead space and both ICU mortality and discharge. The study design was defined in order to deal with both the extremely large variability in the patients’ conditions and the data recorded in the context of the first two waves of the COVID-19 epidemic when most of the Italian ICUs (shortly followed by other countries worldwide) had to deal with a massive surge in their workload. Although this variability likely led to a loss of recorded information, we could assess the possible predictive role of physiologic dead space monitoring using real-world data.

To our knowledge, this is the first study that directly links time-evolving data with outcomes of critically ill patients with COVID-19-associated ARDS, stressing the importance of dead space monitoring in mechanically ventilated patients, which contains additional information to that provided by the sole monitoring of the PaO$_{2}$/FiO$_{2}$ ratio.

## Methods

### Study design

Retrospective, multicentre, and observational study. Data were collected from the MargheritaTre database^29^, an electronic health record (EHR) developed by the Italian Group for the Evaluation of Interventions in Intensive Care (GiViTI) and the Mario Negri Institute for Pharmacological Research, with the objectives of supporting intensive care practitioners in everyday clinical activities and collecting high-quality data for research purposes. The study was conducted according to the Declaration of Helsinki. The experimental protocol received the approval from the Ethics Committee of the Coordinating Center, Comitato Etico Indipendente di Area Vasta Emilia Centro, CE-VAC (protocol code 17164), and each local ethics committee of the hospitals adopting the MargheritaTre software (Comitato Etico ATS Sardegna, Comitato Etico Regionale della Liguria, Comitato Etico Interaziendale A.S.O. SS. Antonio e Biagio e C. Arrigo di Alessandria, Comitato Etico dell’Insubria, Comitato Etico Brianza, Comitato Etico Regionale per la Sperimentazione Clinica della Regione Toscana Area Vasta Centro, Comitato Etico Regionale per la Sperimentazione Clinica della Regione Toscana Area Vasta Nord Ovest, Comitato Etico di Brescia, Comitato Etico Area Pavia-Policlinico San Matteo, Comitato Etico Fondazione IRCCS Istituto Nazionale dei Tumori, Comitato Etico per la Sperimentazione Clinica delle Province di Treviso e Belluno). The informed consent was collected in agreement with national regulations.

### Study population

All adult patients (older than 17 years old) with COVID-19-associated ARDS admitted to ICUs from different Italian hospitals between February 21st, 2020 and March 31st, 2021 were enrolled in this study. Infection from acute respiratory syndrome coronavirus 2 (SARS-CoV-2) was confirmed by reverse transcriptase-polymerase chain reaction (RT-PCR) tests on naso-pharyngeal swabs or lower respiratory tract aspirates.

### Data collection

Age, sex, and comorbidities upon admission to the ICU were recorded for each patient. In addition to baseline data, the following time series were extracted from the MargheritaTre database: systolic blood pressure (SBP, in mmHg), set or measured tidal volume (V$_{\textrm{T}}$, in mL), positive end-expiratory pressure (PEEP, in cmH$_{2}$O), PaO$_{2}$/FiO$_{2}$ ratio as the ratio of arterial oxygen partial pressure (PaO$_{2}$, in mmHg) to fractional inspired oxygen (FiO$_{2}$), and physiologic dead space (DS). Specifically, physiologic dead space was estimated using the Enghoff’s modification of the Bohr’s Equation^30^ as $DS=(PaCO_2-EtCO_2)/PaCO_2$, where PaCO$_{2}$ is the arterial partial pressure of carbon dioxide (in mmHg) and EtCO$_{2}$ is the end-tidal partial pressure of carbon dioxide (in mmHg). For time series of physiological data (SBP, V$_{\textrm{T}}$, PEEP, PaO$_{2}$/FiO$_{2}$, EtCO$_{2}$, PaCO$_{2}$) only sets of observations recorded in the same 1-h time window throughout the ICU stay (since admission to the last available data) were considered valid. Multiple observations recorded in the same hour window were averaged. Patients belonging to the same intensive care unit were clustered to account for possible differences across centers, which may depend on local hospital policies and resources (e.g. breathing circuit configurations, EtCO$_{2}$ monitors, sedation practices, among others).

### Objectives and outcomes

**Figure 3:**
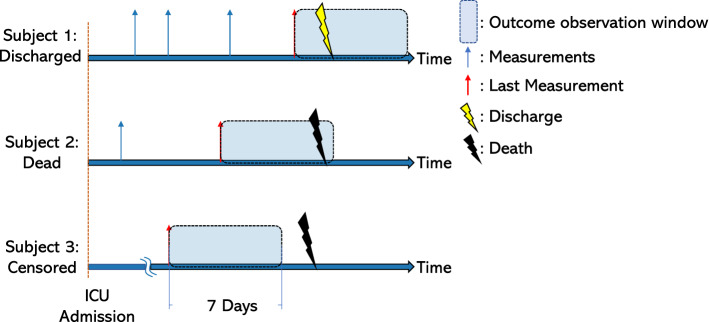
Study design. Each upward-pointing blue arrow represents a set of physiological variables (SBP, V$_{\textrm{T}}$, PEEP, PaO$_{2}$/FiO$_{2}$, EtCO$_{2}$, PaCO$_{2}$), recorded within a 1-h time window. Upward-pointing red arrows represent the last-recorded set of physiological variables. Lightning bolts are the outcomes in study (yellow for discharge from the Intensive Care Unit; black for death in the Intensive Care Unit); only outcomes recorded in a time window of 7 days since the last available measure (blue band) were considered valid for the purpose of the analysis. Subject 1 depicts a patient who was discharged from the Intensive Care Unit within 7 days since the last recorded set of physiologic variables. Subject 2 shows a patient who died within 7 days since the last measured set of data. Subject 3 depicts a patient who experienced the outcome (death) beyond the time window of 7 days since the last recorded set of measurements; data from patients like Subject 3 were censored. *EtCO*$_{2}$ end-tidal partial pressure of carbon dioxide, *PaCO*$_{2}$ arterial partial pressure of carbon dioxide, *PaO*$_{2}$*/FiO*$_{2}$ ratio of arterial oxygen partial pressure (PaO$_{2}$) to fractional inspired oxygen (FiO$_{2}$), *PaO*$_{2}$ arterial oxygen partial pressure, *PEEP* positive end-expiratory pressure, *SBP* systolic blood pressure, *V*$_{\textrm{T}}$ set or measured tidal volume.

The main aim of the study was to investigate the association between physiologic dead space and ICU mortality or ICU discharge within 7 days since the last available measure. Figure 3 graphically depicts the study design.

### Statistical analysis

For a single patient, admission to the ICU can result in two main competing outcomes: death or discharge from the unit. The competing risk modeling approach allows for correctly estimating the marginal probability of an event in presence of competing events. With this perspective, we tested a “Competing Risk Extended Cox Proportional Hazard Model for Time-Dependent Covariates”^31^ to determine the association between the temporal evolution of pulmonary dead space measures and both the patients’ risk of death or probability of discharge. A further description of the proposed modeling approach is provided in the Supplementary material.

#### Model characteristics

The proposed model contains the following features: PaO$_{2}$/FiO$_{2}$ ratio, DS, PEEP, V$_{\textrm{T}}$, SBP, age, sex, presence of diabetes, presence of chronic obstructive pulmonary disease (COPD).

Age and sex were included in the model as they proved to be significant predictors of death in patients with COVID-19-associated ARDS^26^, together with diabetes and COPD^32^, which are highly prevalent conditions associated with pre-existing endothelial damage and pulmonary dysfunction, respectively. SBP was included to correct for the global hemodynamic status. The set or measured tidal volume is a major determinant of minute ventilation and CO$_{2}$ elimination, while PEEP may influence the venous admixture and affect lung recruitment or overdistension^25^. V$_{\textrm{T}}$ and PEEP were used to correct for the effect of ongoing ventilatory support; however, they may also be proxies of the severity of disease, with higher PEEP or lower set V$_{\textrm{T}}$ being used in more critical patients.

PaO$_{2}$/FiO$_{2}$ and DS are widely-recognized predictors of outcome in ARDS^10,33^ and were included to account for the severity of impairment in pulmonary gas exchange arising from intrapulmonary shunt, ventilation/perfusion mismatch and diffusion impairment.

The inclusion of DS was also justified by the observed significant improvement in the performance of the model, as well as by the significant role of DS in predicting both death and discharge, with a relative importance comparable to PaO$_{2}$/FiO$_{2}$ (especially in patients experiencing ICU discharge). These observations strengthened the significant role of DS when correcting for the other confounding variables.

Observations were clustered according to the corresponding patient and ICU. The proportional hazards assumption was tested for all the variables included in the model with a significance level of 0.05.

The likelihood ratio test was used to evaluate whether the inclusion of DS led to a significant improvement in the model performances. The same model was also fitted after normalizing the variables with a Z-score transformation (i.e., subtracting the average value and diving by the standard deviation), in order to have an estimate of the feature importance from the obtained coefficients and hazard ratios, and to provide a more quantitative interpretation of the Hazard Ratios. Categorical variables are reported as frequencies (percentages) and continuous variables as means (with standard deviations, SDs) or medians (with interquartile ranges, IQRs) as appropriate. PostgreSQL 9.5.25, Python 3.8.10, and R 3.6.3 softwares were used to perform the analysis.

## Supplementary Information

Supplementary Information.

## Data Availability

Access to data supporting our findings is restricted to credentialed users only. Access to data can be provided after asking for permission to Dr. Stefano Finazzi (stefano.finazzi@marionegri.it). Interested users will be asked to sign a data use agreement according to European and Italian regulations.
