# Supplementary material for: Physiologic dead space is independently associated with mortality and discharge of mechanically ventilated patients with COVID-19 ARDS: a retrospective study
Source: Sci Rep. 2023 Apr 7;13:5719. doi: 10.1038/s41598-023-31999-6 (PMC10081332; doi:10.1038/s41598-023-31999-6)
Supplement: Supplementary file 1 — Supplementary Information. [file 41598_2023_31999_MOESM1_ESM.pdf]

Supplementary material for: physiologic dead space is independently associated with mortality and discharge of mechanically ventilated patients with COVID-19 ARDS: a retrospective cohort study

Maximiliano Mollura[1,*], Francesca Baroncelli[2], Giulia Mandelli[3], Giovanni Tricella[3], Gary E. Weissman[4], Daniele Poole[5], Pietro Caironi[6,7], Leo Anthony Celi[8,9,10], Riccardo Barbieri[1], and Stefano Finazzi[3,+]

[1]Department of Electronic, Information and Bioengineering, Politecnico di Milano, Milano, Lombardia, Italy
[2]Department of Anesthesia and Intensive Care, San Giovanni Bosco Hospital - ASL Città di Torino, Torino, Piemonte, Italy
[3]Department of Public Health Research, Mario Negri Institute for Pharmacological Research IRCCS, Ranica, 24020, Lombardia, Italy
[4]Palliative and Advanced Illness Research (PAIR) Center and Pulmonary, Allergy and Critical Care Division, University of Pennsylvania Perelman School of Medicine, Philadelphia, Pennsylvania, USA
[5]Operative Unit ofAnesthesia and Intensive Care Unit, S. Martino Hospital, Belluno, Veneto, Italy
[6]Department of Anesthesia and Critical Care, Azienda Ospedaliero-Universitaria S. Luigi Gonzaga, Orbassano, Piemonte, Italy
[7]Department of Oncology, University of Turin, Torino, Piemonte, Italy
[8]Laboratory for Computational Physiology, Massachusetts Institute of Technology, Cambridge, Massachusetts, USA
[9]Division of Pulmonary, Critical Care and Sleep Medicine, Beth Israel Deaconess Medical Center, Boston, Massachusetts, USA
[10]Department of Biostatistics, Harvard T.H. Chan School of Public Health, Boston, Massachusetts, USA
[*]maximiliano.mollura@polimi.it
[+]stefano.finazzi@marionegri.it

ABSTRACT

Background: Physiologic dead space is a well-established independent predictor of death in patients with acute respiratory distress syndrome (ARDS). Here, we explore the association between a surrogate measure of dead space (DS) and early outcomes of mechanically ventilated patients admitted to Intensive Care Unit (ICU) because of COVID-19-associated ARDS.
Methods: Retrospective cohort study on data derived from Italian ICUs during the first year of the COVID-19 epidemic. A competing risk Cox proportional hazard model was applied to test for the association of DS with two competing outcomes (death or discharge from the ICU) while adjusting for confounders.
Results: The final population consisted of 401 patients from seven ICUs. A significant association of DS with both death (HR 1.204; CI 1.019-1.423; $p = 0.029$) and discharge (HR 0.434; CI 0.414-0.456; $p<0.001$) was noticed even when correcting for confounding factors (age, sex, chronic obstructive pulmonary disease, diabetes, PaO_2/FiO_2, tidal volume, positive end-expiratory pressure, and systolic blood pressure).
Conclusion: These results confirm the important association between DS and death or ICU discharge in mechanically ventilated patients with COVID-19-associated ARDS. Further work is needed to identify the optimal role of DS monitoring in this setting and to understand the physiological mechanisms underlying these associations.

This file contains the following supplementary information, supporting the results of the research:

- A detailed mathematical explanation of the competing risk Cox proportional hazard model is provided in page 3.
- Table S1 shows demographic data and outcomes.
- Table S2 shows comorbidities.
- Table S3 shows summary for first and last parameters recorded.
- Table S4 shows the obtained coefficients, standard errors, hazard ratios and p-values for the standardized model.
- Figure S1 shows the evolution of trends in the PaO_2/FiO_2 ratio and Dead Space (DS).

The study was conducted following the criteria provided in the Strengthening the Reporting of Observational Studies in Epidemiology (STROBE) Checklist[1].

Competing Risk Cox Proportional Hazard Model

Cox models are a class of survival models (also known as time to event analyses) whose main goal is to derive a survival function $S(t) = P(T^{(r)} \geq t)$, i.e. the probability that an event r, among R possible events, has not occurred before time t. The defined probability is commonly modeled with an exponential function, where $h(r)$ is the Hazard function for each of the R events:

$$S(t) = \exp\left(-\sum_{r=1}^{R}\int_{0}^{t} h^{(r)}(u)du\right) \tag{1}$$

The Hazard function indeed represents the probability rate of having an event in a specific time window $(t, t+\Delta t)$ where Δt is the duration of the window according to the following formulation:

$$h^{(r)}(t) = \lim_{\Delta t \to 0} \frac{P(t \leq T^{(r)} \leq t + \Delta t | T^{(r)} \geq \Delta t)}{\Delta t} \tag{2}$$

In a Competing Risk Cox Proportional Hazard framework the Hazard function is modeled as follows:

$$h^{(r)}(t, \mathbf{X}) = h_0^{(r)}(t) \exp\left(-\sum_{i=1}^{p} \beta_i^{(r)} \mathbf{X}_i(t)\right) \tag{3}$$

where $h_0^{(r)}(t)$ is the baseline hazard of the r event, $\beta_i^{(r)}$ are the coefficients describing the link between the observations $\mathbf{X}_i$ and the r event.
Therefore, by exponentiating the estimated coefficient $\beta_i^{(r)}$, it is possible to extract the Hazard Ratio ($HR^{(r)} = \exp(\beta_i^{(r)})$) of each variable and for each of the R competing outcomes. A $HR^{(r)} > 1$ indicates an increased probability of experiencing the outcome r for an unitary increase of the corresponding variable while keeping the others constant. Similarly, a $HR^{(r)} < 1$ indicates a reduced probability of experiencing the outcome r, whereas a $HR^{(r)} = 1$ indicates no changes with respect to baseline hazard. Further details on the proposed modeling approach can be found in Austin et al.[2].
The proposed model contains the following features: PaO_2/FiO_2 ratio, DS, positive end-expiratory pressure (PEEP), tidal volume (V_T), systolic blood pressure (SBP), age, sex, presence of diabetes, presence of chronic obstructive pulmonary disease (COPD). Covariates of the model were rescaled for better understanding of the hazard ratios. PaO_2/FiO_2 was scaled by 50 to compute hazards for each 50 mmHg variation. DS, which ranges from 0-100%, was scaled by 10 to show each 10% variation of dead space fraction. V_T was scaled by 10, so that any unitary change in the scaled V_T reflects a 10 mL variation of the tidal volume. PEEP was scaled by a factor of 5, reflecting a 5 cmH_2O variation for each unitary change. SBP was scaled by 20, reflecting a variation of 20 mmHg for any unitary change. Age was scaled by 10, thus reflecting a variation of 10 years for any unitary change.

Cohort Selection and Resulting Population

The initial population included data from distinct Italian ICUs during the first two waves of the COVID-19 epidemic in Italy, in the years 2020 and 2021. Population statistics are similar to those observed in comparable cohorts of critically-ill patients with COVID-19[3,4], which also showed an ICU mortality rate between 30% and 40%, a middle-aged population, and a higher proportion of male patients. Hypertension and diabetes were also among the most prevalent comorbidities[3]. Our cohort selection process consisted of two main steps: the first was the exclusion of ICUs monitoring $EtCO_2$ in less than 50% of patients, while the second excluded patients that did not require invasive ventilation. The former case showed that the starting and the remaining populations had similar characteristics to each other, thus suggesting that the imposed recording requirements, although leading to a strong reduction in the numerosity of the population, did not introduce selection biases. In patients who did not require invasive ventilation, the data on pre-existing medical conditions and ICU outcomes agreed with those expected from a healthier population that did not require invasive ventilatory support, showing a lower mortality rate, a higher discharge rate, and a shorter length of stay.

Ethics Committees The following local ethics committees authorized the data collection process:
Comitato Etico Indipendente di Area Vasta Emilia Centro (CE-AVEC);
Comitato Etico ATS Sardegna;
Comitato Etico Regionale della Liguria ;

Comitato Etico Interaziendale A.S.O. SS. Antonio e Biagio e C. Arrigo di Alessandria;
Comitato Etico dell'Insubria;
Comitato Etico Brianza;
Comitato Etico Regionale per la Sperimentazione Clinica della Regione Toscana Area Vasta Centro;
Comitato Etico Regionale per la Sperimentazione Clinica della Regione Toscana Area Vasta Nord Ovest;
Comitato Etico di Brescia;
Comitato Etico Area Pavia - Policlinico San Matteo;
Comitato Etico Fondazione IRCCS Istituto Nazionale dei Tumori;
Comitato Etico per la Sperimentazione Clinica delle Province di Treviso e Belluno.

Population Statistics Results

Demographic variables, pre-existing conditions and outcomes for the selected populations are shown in Tables S1 and S2. Tables S3a and S3b show summary statistics for the first and last set of physiologic variables recorded in the final population. Overall, a significantly lower median PaO_2/FiO_2 ratio was observed in non-survivors (124; IQR 95-167) than in survivors (162; IQR 124-235; p<0.0001), already in the first set of recordings. This difference was even more marked in the last set of recordings, with a difference of more than 100 units in the median PaO_2/FiO_2 ratio between non-survivors (111; IQR 79-157) and survivors (217; IQR 163-276; p<0.0001). Similarly, the first recorded median physiologic dead space was already significantly higher in non-survivors (33%; IQR, 22-41 %) than in survivors (22%; IQR, 12-34 %; p<0.0001). While the median DS further increased in non-survivors (37%; IQR, 28-44), it slightly decreased in surviving patients in the last recorded set of physiologic data (19%; IQR, 6-30%; p<0.0001). The dynamic course of the PaO_2/FiO_2 and the DS in the final population was followed throughout the ICU stay, and is visually displayed in Figure S1. While no difference was observed between the median V_T in survivors and non-survivors in the first recorded set of physiologic variables (450 mL; IQR, 410-506 mL in non-survivors; 451 mL; IQR, 398-500 mL in survivors; p=0.6201), a significantly lower median tidal volume was measured in non-survivors in the last set of recordings (442 mL; IQR, 372-508) as compared to survivors (480 mL; IQR, 404-570 mL; p=0.0003). Accordingly, while the median PEEP values did not significantly differ between survivors and non-survivors in the first measurements (12 cmH_2O; IQR, 9-14 cmH_2O in survivors; 12 cmH_2O; IQR, 10-14 cmH_2O in non-survivors; p= 0.1531), higher median PEEP requirements were recorded in non-survivors (12 cmH_2O; IQR, 8-14 cmH_2O) than in survivors (10 cmH_2O; IQR, 8-12 cmH_2O; p=0.0001) in the last set of measurements.

	**Starting Population** (n = 1761)	**ICUs with $EtCO_2$**❉ (n = 972)	**MV**† (n = 818)	**No MV**‡ (n = 154)	**Insufficient Recordings** ♦ (n = 361)	**Sufficient Recordings**❉ (n = 457)	**Final Population** (n = 401)
Sex (% Females)	453 (25.7)	232 (23.9)	193 (23.6)	39 (25.3)	88 (24.4)	105 (23.0)	95 (23.7)
Age, years	66.0 (58.0-73.0)	65.0 (56.0-72.0)	66.0 (58.0-72.0)	63.0 (50.0-73.0)	66.0 (58.0-72.0)	65.0 (58.0-71.0)	66.0 (59.0-71.0)
ICU LOS, days	10.0 (4.0-20.0)	11.0 (5.0-22.0)	14.0 (7.0-25.0)	3.0 (2.0-5.0)	9.0 (4.0-18.0)	18.0 (11.0-30.0)	18.0 (10.0-30.0)
Dead (%)	587 (0.33)	278 (0.29)	266 (0.33)	12 (0.08)	96 (0.27)	170 (0.37)	150 (0.37)
Discharged (%)	884 (0.5)	467 (0.48)	353 (0.43)	114 (0.74)	169 (0.47)	184 (0.4)	163 (0.41)
Censored (%)	290 (0.16)	227 (0.23)	199 (0.24)	28 (0.18)	96 (0.27)	103 (0.23)	88 (0.22)

Table S 1. Demographic variables and outcomes for the selected populations. Data are median (interquartile range) or numbers (%). Percentages may not total 100 because of rounding. ❉refers to Intensive Care Units which recorded $EtCO_2$ in more than 50% of patients. †MV indicates patients who received invasive mechanical ventilation (MV), via endotracheal intubation or tracheostomy, during their stay in the Intensive Care Unit. ‡No MV indicates patients who never received invasive mechanical ventilation during their stay. ♦ refers to patients for which insufficient simultaneous recordings were available. ❉ refers to patients for which sufficient simultaneous recordings of physiological measures were available. $EtCO_2$, end-tidal partial pressure of carbon dioxide. ICU, intensive care unit. LOS, length of stay.

	Starting Population (n = 1567)	ICUs with $EtCO_2$* (n = 852)	MV † (n = 715)	No MV ‡ (n = 137)	Insufficient recordings♦ (n = 314)	Final Population (n = 401)	Dead (n = 150)	Discharged (n = 163)	Censored (n = 88)
**No comorbidities**	239 (15.3)	136 (16.0)	107 (15.0)	29 (21.2)	44 (14.0)	63 (15.7)	14 (9.3)	33 (20.2)	16 (18.2)
**Hypertension**	905 (57.8)	502 (58.9)	429 (60)	73 (53.3)	193 (61.5)	236 (58.9)	104 (69.3)	81 (49.7)	51 (58.0)
**Obesity**	477 (30.4)	235 (27.6)	203 (28.4)	32 (23.4)	77 (24.5)	126 (31.4)	46 (30.7)	55 (33.7)	25 (28.4)
**Type II Diabetes,not on Insulin**	260 (16.6)	125 (14.7)	106 (14.8)	19 (13.9)	48 (15.3)	58 (14.5)	24 (16.0)	27 (16.6)	7 (8.0)
**Type II Diabetes, on Insulin**	117 (7.5)	57 (6.7)	51 (7.1)	6 (4.4)	26 (8.3)	25 (6.2)	10 (6.7)	5 (3.1)	10 (11.4)
**Type I Diabetes**	11 (0.7)	4 (0.5)	2 (0.3)	2 (1.5)	1 (0.3)	1 (0.2)	0 (0)	1 (0.6)	0 (0)
**COPD**	133 (8.5)	65 (7.6)	53 (7.4)	12 (8.8)	28 (8.9)	25 (6.2)	7 (4.7)	13 (8.0)	5 (5.7)
COPD, moderate	114 (7.3)	57 (6.7)	48 (6.7)	9 (6.6)	25 (8.0)	23 (5.7)	6 (4.0)	12 (7.4)	5 (5.7)
COPD, severe	19 (1.2)	8 (0.9)	5 (0.7)	3 (2.2)	3 (1.0)	2 (0.5)	1 (0.7)	1 (0.6)	0 (0)
**Asthma**	65 (4.1)	29 (3.4)	26 (3.6)	3 (2.2)	12 (3.8)	14 (3.5)	5 (3.3)	7 (4.3)	2 (2.3)
**Restrictive pulmonary disease**	6 (0.4)	3 (0.4)	3 (0.4)	0 (0)	1 (0.3)	2 (0.5)	0 (0)	2 (1.2)	0 (0)
**Myocardial infarction**	131 (8.4)	78 (9.2)	65 (9.1)	13 (9.5)	34 (10.8)	31 (7.7)	16 (10.7)	12 (7.4)	3 (3.4)
**Arrhythmia**	106 (6.8)	48 (5.6)	38 (5.3)	10 (7.3)	18 (5.7)	20 (5.0)	11 (7.3)	6 (3.7)	3 (3.4)
**CHF (NYHA class 2 or 3)**	40 (2.6)	19 (2.2)	13 (1.8)	6 (4.4)	9 (2.9)	4 (1.0)	2 (1.3)	1 (0.6)	1 (1.1)
**CHF (NYHA class 4)**	1 (0.1)	0 (0)	0 (0)	0 (0)	0 (0)	0 (0)	0 (0)	0 (0)	0 (0)
**Cancer, not metastatic**	54 (3.4)	27 (3.2)	25 (3.5)	2 (1.5)	12 (3.8)	13 (3.2)	6 (4.0)	7 (4.3)	0 (0)
**Hematological disorders**	18 (1.1)	9 (1.1)	9 (1.3)	0 (0)	4 (1.3)	5 (1.2)	2 (1.3)	1 (0.6)	2 (2.3)
**Autoimmune diseases**	31 (2.0)	17 (2.0)	12 (1.7)	5 (3.6)	4 (1.3)	8 (2.0)	4 (2.7)	3 (1.8)	1 (1.1)
**CKD, moderate or severe**	80 (5.1)	41 (4.8)	33 (4.6)	8 (5.8)	18 (5.7)	15 (3.7)	6 (4.0)	8 (4.9)	1 (1.1)
**CKD, terminal**	7 (0.4)	3 (0.4)	1 (0.1)	2 (1.5)	0 (0)	1 (0.2)	1 (0.7)	0 (0)	0 (0)
**Cerebral vascular disease**	54 (3.4)	33 (3.9)	26 (3.6)	7 (5.1)	13 (4.1)	13 (3.2)	7 (4.7)	4 (2.5)	2 (2.3)
**Hemiplegia, paraplegia, tetraplegia**	13 (0.8)	10 (1.2)	8 (1.1)	2 (1.5)	3 (1.0)	5 (1.2)	3 (2.0)	1 (0.6)	1 (1.1)
**Neuromuscular disease**	11 (0.7)	7 (0.8)	4 (0.6)	3 (2.2)	4 (1.3)	0 (0)	0 (0)	0 (0)	0 (0)
**Antiplatelet therapies**	55 (3.5)	18 (2.1)	14 (2.0)	4 (2.9)	7 (2.2)	7 (1.7)	5 (3.3)	2 (1.2)	0 (0)
**Drug-induced coagulopathies**	7 (0.4)	3 (0.4)	3 (0.4)	0 (0)	1 (0.3)	2 (0.5)	1 (0.7)	1 (0.6)	0 (0)
**Coagulopathy**	8 (0.5)	5 (0.6)	2 (0.3)	3 (2.2)	1 (0.3)	1 (0.2)	1 (0.7)	0 (0)	0 (0)

Table S 2. Pre-existing conditions in selected populations. Data are number (%). *refers to Intensive Care Units which recorded $EtCO_2$ in more than 50% of patients. †MV indicates patients who received invasive mechanical ventilation (MV), via endotracheal intubation or tracheostomy, during their stay in the Intensive Care Unit. ‡No MV indicates patients who never received invasive mechanical ventilation during their stay. ♦ refers to patients for which insufficient simultaneous recordings were available. CHF, chronic heart failure. CKD, chronic kidney disease. COPD, chronic obstructive pulmonary disease. NYHA, New York functional classification for heart failure.

	All (n = 405)	Censored (n = 88)	Died (n = 151)	Discharged (n = 166)	p-value
**PaO_2**, mmHg					
Median (IQR)	93 (77-122)	98 (81-125)	90 (73-111)	94 (80-127)	0.0377
**PEEP**, cmH_20					
Median (IQR)	12 (10-14)	12 (10-15)	12 (10-14)	12 (9-14)	0.1531
**FiO_2**, %					
Median (IQR)	70 (55-80)	69 (55-80)	80 (63-90)	65 (50-80)	<0.0001
**$EtCO_2$**, mmHg					
Median (IQR)	35 (30-42)	35 (30-41)	35 (29-42)	36 (30-42)	0.4938
**$PaCO_2$**, mmHg					
Median (IQR)	48 (42-56)	45 (40-54)	51 (44-60)	47 (40-53)	0.0001
**V_T**, mL					
Median (IQR)	451 (402-510)	462 (410-521)	450 (410-506)	451 (398-500)	0.6201
**PaO_2/FiO_2**, mmHg					
Median (IQR)	143 (110-206)	158 (117-214)	124 (97-168)	160 (124-235)	<0.0001
**DS**, %					
Median (IQR)	27 (16-37)	27 (16-33)	33 (22-41)	22 (12-34)	<0.0001
**Lactate**, mmol/L					
Median (IQR)	1 (1-2)	1 (1-2)	1 (1-2)	1 (1-2)	0.0003
**pH**					
Median (IQR)	7.38 (7.31-7.43)	7.37 (7.33-7.43)	7.35 (7.27-7.42)	7.39 (7.34-7.45)	<0.0001

(a) First recorded parameters.

	All (n = 405)	Censored (n = 88)	Died (n = 151)	Discharged (n = 166)	p-value
**PaO_2**, mmHg					
Median (IQR)	91 (74-114)	94 (79-118)	83 (63-102)	95 (80-123)	<0.0001
**PEEP**, cmH20					
Median (IQR)	10 (8-12)	10 (8-12)	12 (8-14)	10 (8-12)	0.0001
**FiO_2**, %					
Median (IQR)	55 (40-78)	50 (40-55)	80 (65-95)	50 (40-60)	<0.0001
**$EtCO_2$**, mmHg					
Median (IQR)	35 (30-42)	34 (30-40)	36 (30-45)	36 (30-41)	0.1102
**$PaCO_2$**, mmHg					
Median (IQR)	47 (40-58)	45 (39-52)	58 (47-73)	42 (38-49)	<0.0001
**V_T**, mL					
Median (IQR)	460 (395-550)	477 (404-580)	442 (372-508)	480 (404-570)	0.0003
**PaO_2/FiO_2**, mmHg					
Median (IQR)	175 (114-247)	204 (153-290)	111 (78-156)	219 (164-276)	<0.0001
**DS**, %					
Median (IQR)	27 (14-38)	25 (14-32)	37 (28-44)	19 (6-30)	<0.0001
**Lactate**, mmol/L					
Median (IQR)	1 (1-2)	1 (1-2)	1 (1-2)	1 (1-2)	<0.0001
**pH**					
Median (IQR)	7.42 (7.34-7.47)	7.41 (7.36-7.46)	7.35 (7.24-7.43)	7.45 (7.42-7.49)	<0.0001

(b) Last recorded parameters.

Table S 3. First (a) and last (b) recorded parameters in the final population. Data are shown separately for the total number of patients with available physiological data, for those who were censored, for those who died or were discharged from the Intensive Care Unit. DS, physiologic dead space. $EtCO_2$, end-tidal partial pressure of carbon dioxide. FiO_2, fraction of inspired oxygen. $PaCO_2$, arterial partial pressure of carbon dioxide. PaO_2, arterial partial pressure of oxygen. PEEP, positive end-exipiratory pressure. V_T, tidal volume. The Kruskal-Wallis test was applied to test for differences between the patients who died or were discharged from the Intensive Care Unit within seven days since the last available set of physiologic data.

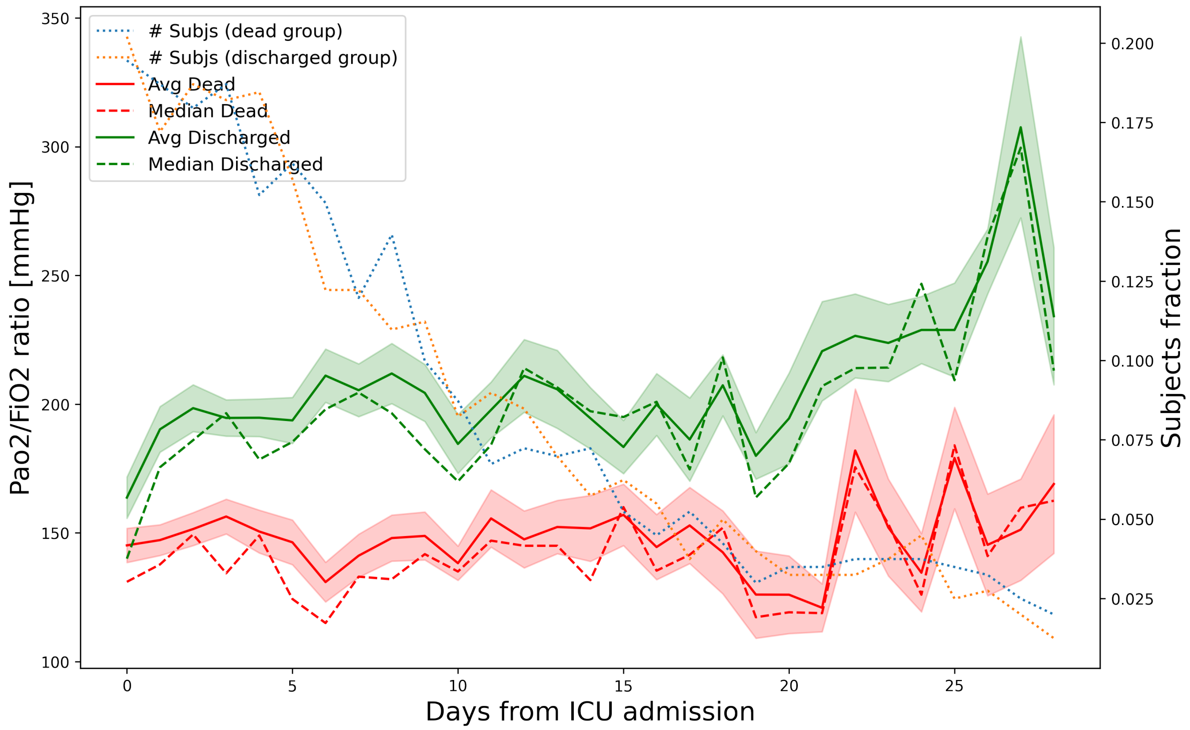

(a) Trend in PaO_2/FiO_2 during the ICU course

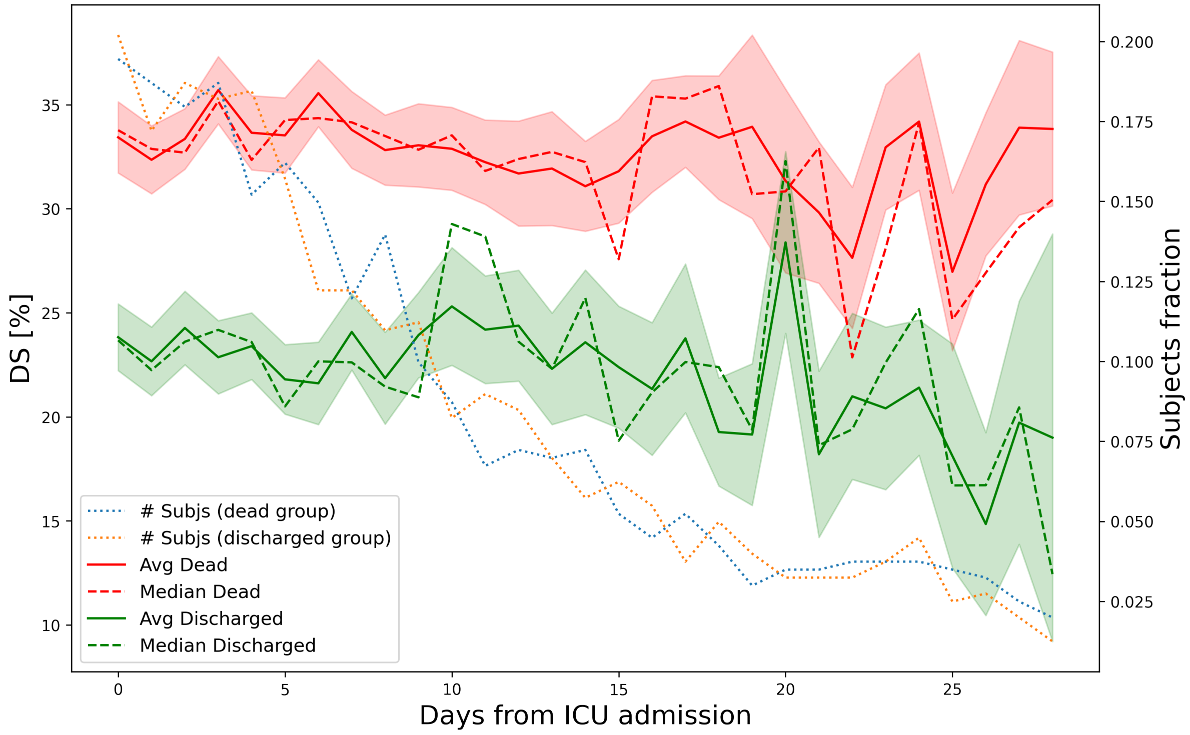

(b) Trend in DS during the ICU course.

Figure S 1. Evolution of trends in the PaO_2/FiO_2 ratio (a) and DS (b) in time. Data for PaO_2/FiO_2 and DS are displayed separately for patients who died in the Intensive Care Unit (red) and patients who were discharged (green). Solid red and green lines are means; dotted red and green lines are medians; the light red and green bands are the standard errors of the mean. Dotted yellow and blue lines show the number of patients considered in the analysis for each day since admission in the Intensive Care Unit, respectively. DS, physiologic dead space measured through the Enghoff's modification of Bohr's equation. ICU, Intensive Care Unit. PaO_2/FiO_2, ratio of arterial oxygen partial pressure (PaO_2, in mmHg) to fractional inspired oxygen (FiO_2).

Feature	Coefficient	Hazard Ratio	SE (coef)	SE robust	Z statistics	p-value
**PaO_2/FiO_2** Death	-1.2526	0.2858	0.2012	0.2294	-5.4594	0.0000
**DS** Death	0.2724	1.3131	0.1397	0.1248	2.1824	0.0291
**V_T** Death	-0.1932	0.8243	0.1435	0.1331	-1.4511	0.1468
**PEEP** Death	0.4410	1.5542	0.1260	0.0969	4.5513	0.0000
**SBP** Death	-0.3768	0.6860	0.1357	0.1027	-3.6705	0.0002
**AGE** Death	0.2681	1.3075	0.1527	0.0978	2.7409	0.0061
**SEX** Death	-0.5109	0.5999	0.3339	0.2375	-2.1512	0.0315
**COPD** Death	0.3983	1.4893	0.5137	0.2497	1.5953	0.1106
**DIAB** Death	0.4490	1.5668	0.3966	0.3144	1.4283	0.1532
**PaO_2/FiO_2** Discharge	0.5356	1.7084	0.1472	0.0537	9.9744	0.0000
**DS** Discharge	-1.2230	0.2944	0.2060	0.0359	-34.0491	0.0000
**V_T** Discharge	0.6135	1.8469	0.1594	0.0940	6.5288	0.0000
**PEEP** Discharge	-0.8859	0.4124	0.2017	0.1240	-7.1451	0.0000
**SBP** Discharge	0.1062	1.1121	0.1587	0.0806	1.3185	0.1873
**AGE** Discharge	-0.1571	0.8546	0.1371	0.1440	-1.0912	0.2752
**SEX** Discharge	0.7758	2.1722	0.3790	0.1925	4.0305	0.0001
**COPD** Discharge	1.1283	3.0905	0.5946	0.2644	4.2678	0.0000
**DIAB** Discharge	-1.6342	0.1951	0.9402	0.2500	-6.5369	0.0000

Table S 4. Results of the competing risk Cox proportional hazard model with standardized variables. Coefficients, hazard ratios (HR), standard errors (SE) and p-values are provided for each feature of the model with respect to the death or discharge outcome at seven days since the last available set of predefined physiological variables. AGE: age in years. COPD: presence or absence of chronic obstructive pulmonary disease. DIAB: presence or absence or diabetes mellitus. DS : physiologic dead space estimated using the Enghoff's modification of the Bohr's Equation. PEEP: positive end-expiratory pressure, in cmH_2O. PaO_2/FiO_2: ratio of arterial oxygen partial pressure (PaO_2, in mmHg) to fractional inspired oxygen (FiO_2). SBP: systolic blood pressure in mmHg. SEX: patient's sex (females = 1, males = 0). V_T: tidal volume, in mL.

References

1. von Elm, E. *et al.* The strengthening the reporting of observational studies in epidemiology (strobe) statement: guidelines for reporting observational studies. *J. Clin. Epidemiol.* **61**, 344–349, DOI: 10.1016/j.jclinepi.2007.11.008 (2008).
2. Austin P.C., L. D. & J.P., F. Introduction to the analysis of survival data in the presence of competing risks. *Circulation* **133**, 601–609 (2016).
3. Grasselli, G. *et al.* Risk Factors Associated With Mortality Among Patients With COVID-19 in Intensive Care Units in Lombardy, Italy. *JAMA Intern Med* **180**, 1345–1355 (2020).
4. Armstrong, R. A., Kane, A. D., Kursumovic, E., Oglesby, F. C. & Cook, T. M. Mortality in patients admitted to intensive care with COVID-19: an updated systematic review and meta-analysis of observational studies. *Anaesthesia* **76**, 537–548 (2021).
